# Valorization of Food Waste: Extracting Bioactive Compounds for Sustainable Health and Environmental Solutions

**DOI:** 10.3390/antiox14060714

**Published:** 2025-06-11

**Authors:** Nikša Bekavac, Korina Krog, Ana Stanić, Dunja Šamec, Anita Šalić, Maja Benković, Tamara Jurina, Jasenka Gajdoš Kljusurić, Davor Valinger, Ana Jurinjak Tušek

**Affiliations:** 1 University of Zagreb Faculty of Food Technology and Biotechnology, Pierottijeva 6, 10000 Zagreb, Croatia; nbekavac@pbf.hr (N.B.); kkrog@pbf.hr (K.K.); astanic@pbf.hr (A.S.); maja.benkovic@pbf.unizg.hr (M.B.); tamara.jurina@pbf.unizg.hr (T.J.); jasenka.gajdos@pbf.unizg.hr (J.G.K.); davor.valinger@pbf.unizg.hr (D.V.); 2 Department of Food Technology, University North, Trg Dr. Žarka Dolinara 1, 48000 Koprivnica, Croatia; dsamec@unin.hr; 3 University of Zagreb Faculty of Chemical Engineering and Technology, Marulićev Trg 19, 10000 Zagreb, Croatia; asalic@fkit.unizg.hr

**Keywords:** food waste valorization, bioactive compounds, sustainable food systems, circular food economy

## Abstract

Food waste is a major economic, environmental, and ethical challenge, as around a third of the edible food produced worldwide is lost or wasted. This inefficiency not only increases food insecurity but also results in resource depletion and environmental degradation. Dealing with food waste through sustainable management strategies, such as upcycling food by-products, has proven to be a promising approach to optimize resource use and support the circular economy. Valorization of food waste enables the extraction of valuable bioactive compounds with strong antioxidant properties. These natural antioxidants play a crucial role in mitigating diseases caused by oxidative stress, including cardiovascular diseases, neurodegenerative diseases, and diabetes. Utilizing food-derived polysaccharides as functional ingredients in the food, pharmaceutical, and cosmetics industries represents an environmentally friendly alternative to synthetic additives and is in line with global sustainability goals. Various extraction techniques, including enzymatic hydrolysis and ultrasound-assisted methods, enhance the recovery of these bioactives while preserving their structural integrity and efficacy. By integrating technological advances and sustainable practices, the food industry can significantly reduce waste while developing high-value products that contribute to human health and environmental protection. This review underscores the significance of food by-product valorization, aiming to bridge the gap between fundamental research and practical applications for a more sustainable future. The literature was selected based on scientific relevance, methodological quality, and applicability to the food, pharmaceutical, or cosmetic sectors. Studies lacking empirical data, not addressing the extraction or application of bioactives, or published in languages other than English were excluded.

## 1. Introduction

Food waste is a significant global issue with economic, environmental, and ethical implications. While food is discarded in many parts of the world, hunger persists elsewhere, highlighting a moral paradox. An estimated one-third of all food produced for human consumption, approximately 1.3 billion tons annually, is either lost or wasted. In calorie terms, one in four calories intended for human consumption is never eaten. In 2018 alone, around 821.6 million people suffered from hunger [1]. Beyond the economic costs, food waste represents a misuse of valuable resources, such as water, land, energy, labor, and capital, and contributes to environmental degradation through greenhouse gas emissions, soil depletion, excessive water use, and biodiversity loss [2]. While some losses in food systems are inevitable, identifying an economically optimal level of loss is challenging due to trade-offs and the difficulty of valuing non-market goods, such as clean air or biodiversity [3]. Addressing these inefficiencies is a complex yet urgent task for policymakers, researchers, and stakeholders across the agri-food sector [4].

**Figure 1:**
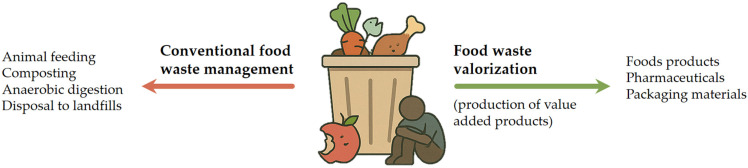
Food waste management.

Global trends such as urbanization, population growth, and pandemics are increasing pressure on food systems. Reducing and repurposing food waste is widely recognized as a key strategy to improve resource efficiency, enhance food security, and support the transition to a sustainable, circular economy. The circular economy model in food systems promotes waste minimization, reuse of by-products, and lifecycle extension of food-derived materials through innovative processing and upcycling. These efforts align with the United Nations Sustainable Development Goals, particularly Goal 12.3, which aims to halve global per capita food waste by 2030 [5]. Amid rising food prices and widespread food insecurity, the loss of nutrient-rich materials further exposes systemic inefficiencies. Developing sustainable food waste management strategies, such as bioconversion, enzymatic extraction, and the production of high-value compounds, can reduce waste while supporting environmental and social goals (Figure 1) [6]. The image contrasts conventional food waste management methods, such as animal feeding, composting, and landfill disposal, with the more sustainable approach of food waste valorization. It highlights how by-products can be transformed into value-added products like foods, pharmaceuticals, and packaging materials, promoting a circular economy.

Upcycling food by-products presents a promising solution by transforming waste into valuable ingredients for industrial use. The food industry generates substantial volumes of by-products, including fruit and vegetable peels, seeds, stems, and pulp, many of which are discarded despite being rich in bioactive compounds such as antioxidants, fiber, proteins, and essential fatty acids. Utilizing these by-products reduces the environmental impact, conserves resources, and creates innovative products for the food, pharmaceutical, and cosmetic sectors [7]. A key advantage of upcycled by-products is their high antioxidant content. Peels, seeds, and leaves of fruits and vegetables contain phenolic compounds, flavonoids, carotenoids, and vitamins C and E, which are effective in neutralizing free radicals and reducing oxidative stress. These compounds are known to help prevent chronic diseases, such as cardiovascular conditions, cancer, and neurodegenerative disorders [8,9,10]. Extracting natural antioxidants from food waste offers a clean-label, sustainable alternative to synthetic additives for use in dietary supplements and functional foods. These extracts can enhance product shelf life, stability, and consumer health benefits [11,12].

**Figure 2:**
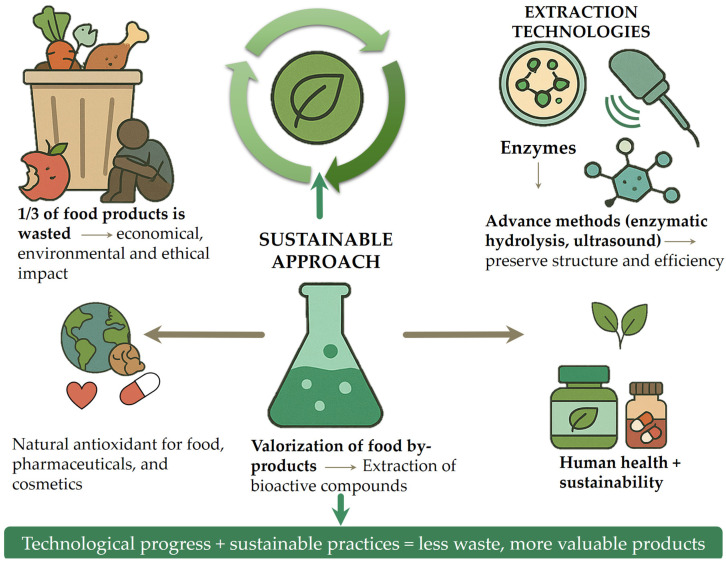
Sustainable approach for the valorization of food by-products through advanced extraction technologies.

Although food waste valorization has been widely recognized as a promising strategy, the existing literature often lacks a focused and up-to-date synthesis that connects recent technological advances with their practical applications in industry. This review addresses that gap by consolidating peer-reviewed research published primarily between 2010 and 2024, with an emphasis on sustainable extraction techniques and the recovery of high-value bioactive compounds, particularly natural antioxidants and polysaccharides, from food by-products. A key contribution of this work is its multidisciplinary perspective, linking bioactive compound recovery not only to their chemical properties but also to their cross-sector applications in the food, pharmaceutical, and cosmetic industries. Unlike broader or purely theoretical discussions, this review frames food waste valorization within the context of the circular economy and the United Nations’ Sustainable Development Goal 12.3, offering a policy-relevant and application-oriented synthesis. Special attention is given to bioactive-rich materials, such as peels, seeds, and pulp, which can serve as sustainable, clean-label alternatives to synthetic additives in health-promoting products. By evaluating both the scientific foundations and industrial-scale implementation of these strategies, the review aims to bridge the gap between research and practice, demonstrating how food waste can be transformed into valuable resources that support both environmental sustainability and human health. Figure 2 illustrates a sustainable strategy for food by-product valorization, highlighting the integration of advanced extraction technologies designed to maximize the recovery of functional compounds. The literature was selected based on scientific relevance, methodological rigor, and applicability to target industries; studies lacking empirical data, those not addressing bioactive extraction or application, and non-English publications were excluded.

## 2. Bioactive Compounds in Food Waste and Their Health Benefits

**Figure 3:**
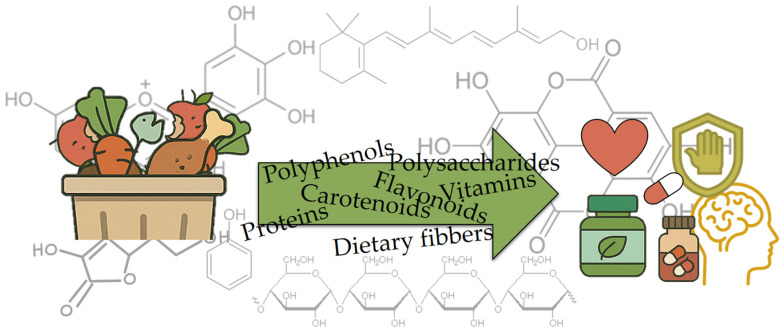
Bioactive compounds in food waste.

Recently, the shift toward sustainable development has led to the valorization of waste products from the food industry as a source of high-value “functional ingredients“ [13]. Bioactive compounds in food waste are gaining increasing attention due to their potential health benefits, as well as their ability to reduce environmental pollution and increase food sustainability. Food waste, especially from fruits and vegetables, is often rich in bioactive compounds such as polyphenols, carotenoids, vitamins, and dietary fibers [11,12,14,15]. Figure 3 depicts the transformation of food waste biomass into a spectrum of high-value bioactives, polyphenols, polysaccharides, carotenoids, flavonoids, vitamins, proteins, and dietary fibers, symbolized by the green arrow overlaying the molecular structures. These recovered compounds are then linked to health-oriented applications, as shown by icons representing nutraceutical supplements, cardiovascular and immune support, and cognitive benefits. These compounds exhibit antioxidant, anti-inflammatory, antimicrobial, and anti-cancer properties that contribute to various health benefits [16,17,18,19,20]. Utilizing bioactive compounds from food waste not only increases the value of discarded food but also represents a sustainable method of resource recovery.

### 2.1. Techniques Used for the Bioactives Extraction

Innovative extraction methods utilizing green solvents for the recovery of bioactive compounds from food waste have received considerable attention in recent years. Conventional extraction techniques, such as maceration, Soxhlet extraction, and solvent extraction with petroleum-based solvents, are often inefficient, consume a lot of energy, take a long time, and are harmful to the environment. In contrast, innovative extraction techniques, such as supercritical fluid extraction (SFE), subcritical water extraction (SWE), ultrasound-assisted extraction (UAE), microwave-assisted extraction (MAE), and deep eutectic solvent (DES) extraction, offer numerous advantages in terms of efficiency, sustainability, and selectivity [21,22]. Table 1 provides a comparative overview of the conventional and recent extraction techniques used for recovering bioactive compounds from food waste, highlighting their principles, efficiencies, and sustainability aspects.

One of the most promising methods, supercritical fluid extraction (SFE), especially using supercritical CO_2_, offers a non-toxic and environmentally friendly alternative for extracting bioactive compounds. The tunable properties of supercritical CO_2_ allow for selective extraction, reducing the need for additional purification steps. This technique works at relatively low temperatures and preserves the integrity of heat-sensitive bioactive compounds such as polyphenols, flavonoids, and carotenoids. Additionally, CO_2_ is easily recyclable, making this method highly sustainable compared to classic solvent-based techniques. Subcritical water extraction (SWE) is another environmentally friendly alternative in which water is used at elevated temperatures and pressures to modify its dielectric properties and thereby improve its solvating ability for a wide range of bioactives. In contrast to classical extraction methods based on organic solvents, SWE avoids toxicity issues and offers rapid extraction with high yields. This method is particularly effective for extracting phenolic compounds from food waste, like fruit peels and vegetable residues, which are often discarded despite their high antioxidant content. For example, Lachos-Perez et al. [23] demonstrated that subcritical water extraction (SWE) from defatted orange peel yielded 31.70 mg GAE/g of polyphenols, significantly higher than Soxhlet extraction (7.75 mg GAE/g), shaker extraction using ethanol (4.42 mg GAE/g), and ultrasound-assisted extraction with ethanol (5.83 mg GAE/g). Ultrasound-assisted extraction (UAE) and microwave-assisted extraction (MAE) have also proven to be efficient and sustainable alternatives. In UAE, the cell walls are broken up with ultrasonic waves, which facilitates the release of intracellular bioactives. This process is faster, requires less solvent, and enhances mass transfer compared to classic maceration. Similarly, MAE utilizes microwave energy to rapidly heat solvents and plant matrices, significantly reducing the extraction time and improving bioactive yields while consuming less energy than conventional methods. Overall, these innovative extraction methods outperform classical techniques by offering higher efficiency, improved selectivity, lower energy consumption, and reduced environmental impact [24,25]. They align with the principles of green chemistry and the circular economy and are, therefore, ideal for valorizing food waste to high-value bioactive compounds for nutraceutical, pharmaceutical, and cosmetic applications.

### 2.2. Polyphenols

**Figure 4:**
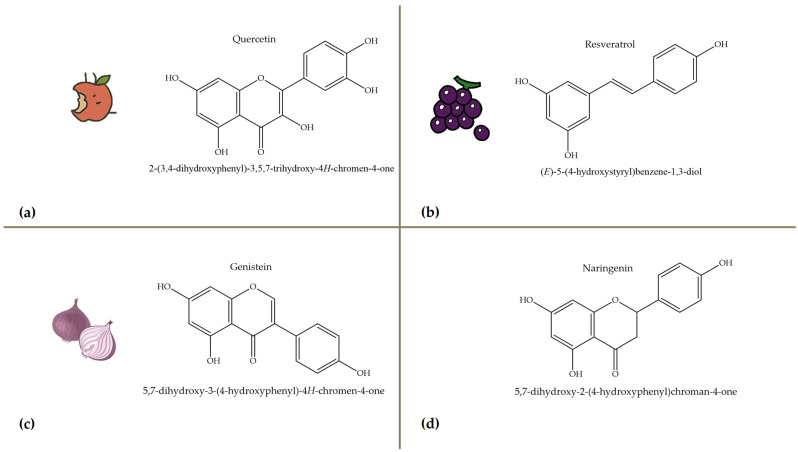
Chemical structures and natural sources of selected polyphenolic compounds. (**a**) Quercetine (from apples); (**b**) resveratrol (from grapes); (**c**) genistein (from onions); (**d**) naringenin (from citrus fruits).

**Figure 5:**
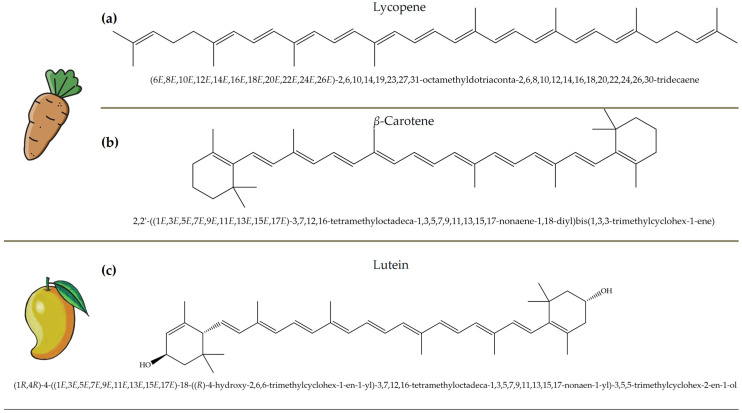
Chemical structures and natural sources of selected carotenoids. (**a**) lycopene (commonly found in carrots and tomatoes); (**b**) β-Carotene (from carrots and other orange vegetables); (**c**) lutein (abundant in mangoes and leafy greens).

Polyphenols, bioactive compounds present in fruit and vegetable waste, are known for their strong antioxidant properties [26]. These compounds play a crucial role in reducing oxidative stress, fighting inflammation, and preventing chronic diseases, making the recovery of these compounds from food waste an important strategy for both human health and sustainability. Apple peels, for example, which are often discarded during processing, are particularly rich in quercetin, a flavonoid known for its anti-inflammatory, anti-proliferative, and cardioprotective effects [27]. Quercetin is associated with a lower risk of heart disease, cancer, and neurodegenerative disorders due to its ability to neutralize free radicals and modulate cellular signaling pathways. Apple peels also contain other polyphenols such as catechins and chlorogenic acid, which contribute to their high antioxidant capacity. Another important polyphenol, resveratrol, is found in grape skins and pomace, which are often discarded during wine production [28]. Figure 4 focuses on selected polyphenols, including quercetin, resveratrol, genistein, and naringenin, derived from fruits and vegetables, while Figure 5 depicts representative carotenoids, such as lycopene, β-carotene, and lutein, which are found in commonly consumed plant sources. Resveratrol has been extensively studied for its cardioprotective properties [29], as it helps to lower LDL (bad cholesterol), reduce blood clotting, and improve endothelial function. It also has anti-aging effects by activating sirtuins [30], which are proteins that play a key role in cell repair and longevity.

Similarly, citrus by-products, including orange peel and pulp residues, are an important source of flavonoids, particularly hesperidin and naringenin. These bioactive compounds have been extensively studied for their ability to lower cholesterol, improve blood circulation, and protect against cardiovascular disease [31,32]. In particular, research has demonstrated that the antioxidant activity in citrus peel is significantly higher than in the fruit itself, highlighting its potential as a valuable but underutilized resource for functional food and nutraceutical applications [33,34,35]. For example, Azman et al. [36] investigated the antioxidant properties, such as phenolic and flavonoid content, of fresh and frozen citrus peels. Frozen citrus peels had a significantly higher antioxidant content compared to fresh peels. Frozen lemon peels had a 54.7% higher total flavonoid content than fresh lemon peels. A possible reason for this could be the deactivation of oxidative and hydrolytic enzymes during the freezing process. The obtained results indicate that frozen citrus peels can be used for the production of food supplements due to their exceptional antioxidant activity. Furthermore, the onion (*Allium cepa* L.) is one of the most widely used vegetables in the world, and its processing generates significant amounts of by-products, especially from the skins. Although the skins are often treated as waste due to their intense odor, research has proven that they contain high concentrations of bioactive compounds. Chernukha et al. [37] investigated the different antioxidant profiles of husk waste obtained from red, yellow, and white varieties of onions. Isoflavones, flavonols, flavanonols, and flavonoid-O-glycosides were detected in the skins of red and yellow onions, while the skins of white onions contained lower amounts of antioxidants. Beyond their health-promoting properties, polyphenols extracted from food waste have promising applications in the food, pharmaceutical, and cosmetic industries. Their strong antioxidant and antimicrobial activities make them ideal natural preservatives that extend the shelf life of perishable goods while reducing reliance on synthetic additives. Additionally, polyphenol-rich extracts from fruit and vegetable waste are being incorporated into dietary supplements, skincare formulations, and biodegradable packaging materials, further strengthening their role in sustainability and circular economy practices [22]. By utilizing the full potential of polyphenols from food by-products, the industry can not only contribute to waste reduction but also create value-added products that support human health and environmental protection. Examples of the antioxidant activity of some polyphenols and flavonoids extracted from food waste are given in Table 2.

### 2.3. Carotenoids

Carotenoids, which are abundant in food waste, such as carrot peels, tomato skins, and mango peels, are a valuable class of bioactive compounds with significant health benefits [43]. These naturally occurring pigments have strong antioxidant properties and help to neutralize free radicals, reduce oxidative stress, and protect against chronic diseases. Lycopene, an important carotenoid in tomato skin, has been associated with a reduced risk of prostate and breast cancer due to its strong antioxidant and anti-inflammatory activity [44,45,46]. Studies have shown that lycopene can effectively inhibit lipid peroxidation and DNA damage, making it an important compound for disease prevention and overall health. Similarly, β-carotene is found in high concentrations in carrot peels [47,48]. It serves as a precursor to vitamin A, which is essential for vision, immune function, and skin health. Research indicates that the β-carotene content in carrot peels is about 50% higher than in the pulp, emphasizing the untapped nutritional potential of food waste. This compound not only supports eye health by preventing age-related macular degeneration, but also enhances skin repair and immune response [49]. Figure 5 depicts representative carotenoids, including lycopene, β-carotene, and lutein, found in commonly consumed plant sources.

Mango peels, another rich source of carotenoids such as lutein and zeaxanthin, offer additional health benefits [50,51]. These compounds are particularly beneficial for eye health, as they accumulate in the retina and help protect against harmful blue light and oxidative stress, reducing the risk of cataracts and age-related macular degeneration. Their powerful antioxidant activity also contributes to cardiovascular health by lowering oxidative damage to lipids and improving blood vessel function. Beyond their health-promoting properties, carotenoids are also used industrially in food, cosmetics, and pharmaceuticals. Their natural pigmentation makes them valuable colorants in food and offers a sustainable alternative to synthetic dyes. Their ability to prevent oxidative degradation also makes them ideal natural preservatives that extend the shelf life of food and cosmetic products. Examples of the antioxidant activity of some carotenoids from food waste are given in Table 3.

### 2.4. Dietary Fibers

Dietary fibers, the indigestible parts of plants, are widely recognized for their crucial role in maintaining human health [62,63]. Although commonly associated with whole grains, fruits, and vegetables, a significant amount of these valuable nutrients is often discarded as food waste [64,65]. This waste, consisting of peels, seeds, pulps, and other by-products of food processing and consumption, presents a unique opportunity to recover and utilize these dietary fibers, not only reducing waste but also contributing to improved public health. Dietary fibers are broadly divided into two categories based on their solubility in water: soluble and insoluble [66]. Soluble fibers, such as pectins, gums, and beta-glucans, dissolve in water and form a gel-like substance. Thanks to this property, they can slow down digestion, regulate blood sugar levels, and lower cholesterol levels by hindering the absorption of cholesterol in the intestine. Insoluble fibers, including cellulose and hemicellulose, do not dissolve in water and are primarily used to bulk up stool, promote regular bowel movements, and prevent constipation. Both types of fiber play different but complementary roles in maintaining digestive health and overall well-being.

The health benefits extend beyond their role in digestion. There is much evidence to suggest that an adequate intake of dietary fiber is associated with a lower risk of several chronic diseases [67,68]. For example, the ability of soluble fiber to lower cholesterol levels contributes to improved cardiovascular health and reduces the risk of heart disease, stroke, and other related conditions. Furthermore, the regulation of blood sugar levels by soluble fiber is particularly beneficial for people who have or are at risk of developing type 2 diabetes. By slowing down the absorption of sugar, they help to prevent a rise in blood glucose levels, improve insulin sensitivity, and reduce stress on the pancreas. Insoluble fibers make an equally important contribution to digestive health [69,70]. By adding bulk to the stool, it facilitates the passage of stool through the digestive tract, prevents constipation, and reduces the risk of associated complications, such as hemorrhoids and diverticulosis. Moreover, a high-fiber diet is associated with a lower risk of colorectal cancer [71], possibly due to the shorter transit time of stool through the colon, which minimizes exposure to potential carcinogens. In addition to these well-known benefits, new research suggests that dietary fibers may also play a role in weight control [72,73,74]. High-fiber foods tend to be more filling, which promotes satiety and reduces the likelihood of overeating. Additionally, fermentation of some dietary fibers in the gut produces short-chain fatty acids, which have been shown to have beneficial effects on metabolism and appetite regulation.

Given the abundance of dietary fiber in food waste, recovering and recycling it is a sustainable approach to increasing fiber intake and promoting public health. Various methods exist to extract and purify these fibers from food waste, including physical, chemical, and enzymatic processes [64]. The extracted fibers can then be incorporated into a variety of food products, such as baked goods, cereals, and beverages, enhancing their nutritional value and thus contributing to a more balanced diet.

### 2.5. Vitamins

Although often overlooked, food waste can be a surprisingly rich source of various vitamins. It offers a potential pathway for nutrient recovery and contributes to improved public health and a more sustainable food system [75]. This exploration will delve into the presence of vitamins in food waste, emphasizing their individual health benefits and the potential for their recovery and utilization. Vitamins are organic compounds that are required in small amounts for various physiological functions. They are broadly classified into two categories: fat-soluble vitamins (A, D, E, and K) and water-soluble vitamins (B vitamins and vitamin C). Each vitamin plays a unique and crucial role in maintaining human health, and their deficiency can lead to a range of health problems. Food waste, i.e., peels, seeds, pulps, and other by-products of food processing and consumption, often contains significant concentrations of these vital nutrients. A short overview of the health benefits of the vitamins extracted from food waste is given in Table 4.

The potential for recovering vitamins from food waste is significant. Various extraction methods, including solvent extraction, supercritical fluid extraction, and enzymatic methods, can be used to isolate and concentrate these valuable nutrients. The recovered vitamins can then be incorporated into food supplements or even animal feed, increasing nutritional value and reducing waste. The recovery of vitamins from food waste offers several advantages. It provides a sustainable approach to increasing vitamin intake and reduces reliance on synthetic vitamin supplements. Furthermore, the vitamins contained in food waste can be in a form that is more bioavailable than synthetic vitamins, meaning that they can be more easily absorbed and utilized by the body [88].

### 2.6. Polysaccharides

Polysaccharides, including pectins, hemicelluloses, and dietary fibers, are abundant in food waste such as fruit peels, vegetable residues, and cereal by-products [89]. Various extraction techniques, such as hot water extraction, enzymatic hydrolysis, ultrasound-assisted extraction, and microwave-assisted extraction, are used to efficiently recover these bioactive compounds. The choice of extraction method has an impact on the yield, structural integrity, and bioactivity of the polysaccharides. Among these methods, green and environmentally friendly extraction approaches, such as enzyme-assisted and ultrasound-assisted techniques, are preferred, as they minimize the use of harsh chemicals and maintain the functional properties of the polysaccharides.

One of the most significant properties of polysaccharides extracted from food waste is their potent antioxidant activity. These polysaccharides exhibit antioxidant potential based on mechanisms such as free radical scavenging, metal ion chelation, and inhibition of lipid peroxidation [90]. The presence of hydroxyl groups and other functional moieties in their structure contributes to their electron-donating ability, effectively neutralizing reactive oxygen species. Studies have shown that polysaccharides derived from food waste, such as citrus peels, apple pomace, and mushroom residues, possess high antioxidant capacity comparable to conventional antioxidants such as vitamin C and tocopherols [90,91,92,93]. The molecular weight, degree of branching, and monosaccharide composition significantly influence their antioxidant activity. Furthermore, structural modifications such as sulfation, acetylation, and carboxymethylation can enhance their bioactivity, making them more effective in oxidative stress management. These bioactive compounds contribute to reducing oxidative stress-related diseases, including cardiovascular disease, neurodegenerative diseases, diabetes, and certain cancers. By scavenging free radicals and reducing inflammation, polysaccharides help protect cells from oxidative damage, which is a key factor in aging and chronic disease progression [92,93]. For instance, pectins extracted from fruit peels have been shown to lower cholesterol levels, improve gut health, and modulate the immune system. Similarly, β-glucans from cereal bran and fungal waste enhance immune function by stimulating macrophage activity and cytokine production. Moreover, polysaccharides contribute to gut health by acting as prebiotics, promoting the growth of beneficial gut microbiota [94,95,96,97]. They serve as fermentable substrates for probiotic bacteria, such as *Lactobacillus* and *Bifidobacterium*, leading to the production of short-chain fatty acids (SCFAs) such as butyrate, which play a crucial role in maintaining gut integrity and reducing inflammation. This gut-modulating effect is particularly beneficial for preventing metabolic disorders such as obesity and type 2 diabetes. Additionally, polysaccharides have anti-diabetic properties by regulating blood glucose levels, enhancing insulin sensitivity, and inhibiting carbohydrate-digesting enzymes.

### 2.7. Essential Oils

Plants produce essential oils (EOs), which are volatile aromatic compounds historically used as flavoring agents in food, medicine, and cosmetics. Essential oils are often found in the parts of plants that are considered food waste, such as peels, making these by-products valuable sources for extracting aromatic and bioactive compounds [85]. A good example is citrus fruit peels, whose EOs are primarily composed of a mixture of volatile compounds, including terpenes and oxygenated derivatives, such as aldehydes (like citral), alcohols, and esters [98]. For example, according to the Grover et al. [99], citrus essential oil (EO) is an eco-friendly, inexpensive, and natural alternative to artificial antioxidants and preservatives, with wide applications in enhancing the shelf life of foods like fruits, vegetables, dairy, bakery, and meat products, as well as in the pharmaceutical and cosmetic industries. Conventional methods for extracting essential oils (EOs), such as cold pressing, solvent extraction, and distillation, often require high energy, long processing times, and high temperatures that can degrade heat-sensitive compounds, and sometimes involve toxic solvents unsuitable for food use. As a result, greener technologies are emerging, offering eco-friendly, energy-efficient, and low-emission alternatives for EO extraction [99]. Methods such as supercritical fluid extraction (SFE) [100], Ohmic heating-assisted extraction/hydrodistillation [101], ultrasound-assisted extraction [102], and green and solvent-free extraction processes using ultrasound and microwave techniques [103] have been optimized and developed for EO extraction from citrus peels. According to the review article by Teigiserova et al. [104], solvent-free microwave extraction is the most effective method, which provides a high yield in a short extraction time.

EOs are high in terpenoid compounds, which may also be directly extracted from food waste. For example, the main constituent of orange and other citrus fruit essential oils is d-limonene or (+)-limonene, comprising 3.8–5.3% of waste orange peel and 90–98% of orange essential oil [105]. It is commonly used as a flavoring in food, cosmetics, and medicines, as well as a fragrance in perfumes, with potential medical applications like gallstone dissolution and cancer prevention, and industrial uses in adhesives, solvents, electronics, and lipid extraction from foods [105]. In addition to limonene, orange waste, pine substrates, and apple pomace waste may serve as valuable sources of terpenes, such as camphene, myrcene, p-cymene, terpinolene, cadinene, sabinene, longifolene, germacrene, linalool, terpinene, β-phellandrene, 3-carene, spathulenol, and α-farnesene [18]. According to the review article by Siddiqui et al. [105], for terpenoid extraction, in addition to substituting the conventional solvent hexane with certain bio-based solvents, a range of techniques have been developed. These include enhanced solvent extraction processes through temperature and pressure intensification or ultrasound, improved distillation methods, most commonly using various microwave-based techniques, as well as enzymes and supercritical CO_2_ extraction.

### 2.8. Other Compounds

Cocoa bean shells are industry by-products rich in bioactive compounds, including alkaloids that can be effectively extracted. For instance, cocoa bean shells may serve as a source of theobromine and caffeine, which can be successfully extracted using deep eutectic solvents in combination with microwave-assisted extraction [106]. According to Santonocito et al. [107], food waste of tomato processing may be a source of steroidal alkaloids, which inhibit neuroblastoma cell viability. Also, potato peel waste is a source of steroidal alkaloids, which Hossain et al. [108] successfully extracted by ultrasound-assisted extractions. Sweet pepper stalks, a common food waste, contain alkaloids that exhibit antioxidant and antimicrobial properties [109].

Glucosinolates are sulfur-containing compounds found in cruciferous vegetables that act as precursors to bioactive compounds, such as isothiocyanates and indoles, known for their potential health benefits, including antioxidant, anti-inflammatory, anticancer, and detoxifying properties. For example, Amofa-Diatuo et al. [110] successfully extracted isothiocyanates from cauliflower food waste (leaves and stems) using ultrasound-assisted extraction, and subsequently used these extracts to create new functional apple beverages enriched with isothiocyanates. Glucosinolates are also present in broccoli, cabbage, and other cruciferous vegetable waste, such as leaves and stalks, making them valuable sources of glucosinolates (reviewed by Shinali et al.).

Food waste may also contain other compounds whose potential utilization is uncertain, requiring further research to assess their toxicity and explore their possible pharmaceutical applications. For example, oxygen heterocyclic compounds (OHCs), including coumarins, furocoumarins, and polymethoxyflavones, are specialized plant metabolites that play a vital role in plant defense by protecting against infections and supporting growth. Recent research has extensively documented their presence in various foods and food waste, particularly in citrus fruits, cinnamon, carrots, parsley, and similar items. While the beneficial effects of polymethoxyflavones on human health are well-established, concerns have been raised regarding the potential risks associated with furocoumarins and coumarin, and their presence in extracts has to be monitored. In general, when extracting bioactive compounds from food waste, it is crucial to consider the safety profile, as the waste may contain undesirable compounds that could also be present in the extracts. These unwanted substances could pose potential health risks, highlighting the importance of thorough purification and safety assessments during the extraction process.

## 3. Valorization the Food Waste in the Food Industry

As previously mentioned, food waste and by-products are abundant in numerous bioactive compounds and dietary fibers, which could be used in improving the nutritive value of existing and new food products [25,111,112]. The term “by-product” is increasingly becoming the focus of discussions on the valorization of residues from the food industry. This shift reflects the growing awareness of the potential to utilize these residues as substrates for the production of functional compounds and the development of new products with additional market value. The overarching goal of the industry is, therefore, to reduce food waste, valorize by-products, and improve waste management [113]. Nowadays, most of the food waste that is being valorized is used for food supplements [114]. The substitution of synthetic supplements and food additives with natural alternatives is a growing trend, driven by increasing consumer awareness of product safety and health concerns [115]. Synthetic antioxidants and preservatives have been favored in the food industry due to their purity, availability, consistent activity, and lack of negative impacts on the sensory and physical properties of the final products [116]. However, their artificial origin has led to increased consumer aversion, as many associate them with harmful chemicals. In addition, some synthetic additives can have toxic effects when used in high concentrations, which raises additional health concerns [117]. In the current market, there is a clear trend towards green formulations, where natural preservatives and antioxidants are used not only in food but also in personal care, cosmetics, and pharmaceuticals [118]. Given the serious and growing environmental problem that food waste poses, it is essential to develop sustainable methods for its valorization, such as using food waste as a source of bioactive compounds to create high-value products, whether in the form of entirely new products used as dietary supplements or by adding such food waste to extend the shelf life and improve the nutritional value of existing products [119]. Food waste is often abundant in biologically active compounds, including polyphenols [120], carotenoids [121], dietary fibers [89], carbohydrates [122,123], and proteins [124], each of which has significant potential. Polyphenols are potent antioxidants that help prevent lipid oxidation, thereby extending shelf life and enhancing product stability. Carotenoids offer both antioxidant activity and natural coloring properties, aligning with the clean-label trend. Dietary fibers contribute to gut health and can improve the texture and moisture retention in baked goods. Carbohydrates, particularly indigestible ones, support prebiotic functions and contribute to energy metabolism. Proteins from food waste, such as whey or oilseed cakes, can serve as valuable nutritional enhancers and functional agents (e.g., emulsifiers or foaming agents). Generally, these compounds reflect their multifunctionality, applicability across various food matrices, and strong consumer interest in natural, health-promoting ingredients. In order to reuse food waste efficiently and sustainably, new technologies for the preservation of these compounds need to be explored. Nowadays, food waste is usually used for the production of animal feed, cosmetics, and pharmaceuticals. Stabilization processes are crucial for the successful reuse of food waste, as its high moisture content can lead to the spoilage and degradation of bioactive compounds [125]. Drying, freeze-drying, or dehydration are common methods to reduce the moisture content and preserve the functional properties of the compounds [126]. These processes ensure that the by-products can be stored for longer, and their nutritional and functional integrity is maintained when they are added to food. Examples of some applications of food waste in food products are given in Table 5.

The main food waste of interest is waste from fruit processing. By-products of the fruit-processing industry, such as peels, seeds, and pulp, contain valuable compounds like pectin, flavonoids, carotenoids, fibers, and polyphenols [191,192]. These by-products, typically considered waste, have the potential to be repurposed either by being added to other food products after undergoing stabilization processes, as they often have a high moisture content [193], or by serving as substrates for the extraction of bioactive compounds [194], which can then be used as functional additives in food [195]. Pectin is widely used in the food industry as a gelling agent, particularly in jams and jellies, and also has prebiotic properties that support gut health [196]. Apple pomace and citrus peels are the primary plant materials used for the extraction of pectin, which has a wide range of applications [197,198]. It is commonly used in the production of jams, jellies, and confectionery, but its applications extend beyond food. Pectin’s ability to form gels in acidic environments also makes it useful in pharmaceuticals and cosmetics, where it can serve as an emulsifier or stabilizer [199]. Another use of fruit peels, specifically citrus peels, which are rich in flavonoids and vitamin C, can be processed into powders or extracts and used as natural preservatives or flavor enhancers in a variety of food applications [200,201]. Significant quantities of citrus by-products are promising for the development of health-promoting foods by incorporating them as functional ingredients. Their high content of bioactive compounds, including flavonoids and indigestible carbohydrates such as dietary fibers, makes them ideal for enhancing foods with additional health benefits. In addition to the peels, the seeds are also a promising source of bioactive compounds [202]. Extracts from grapefruit seeds are marketed as a natural and environmentally friendly pesticide in organic farming and have proven their effectiveness against various pathogens. In a study by Kim et al. [203], the electrostatic coating of alginate–chitosan with grapefruit seed extract was tested on shrimps (*Litopenaeus vannamei*). This showed that the shelf life can be extended when stored for 15 days at 4 °C. This highlights the application of citrus seed extracts, not only in crop protection but also in the food industry, particularly in enhancing the quality and safety of seafood products [203]. In addition to grapefruit seed extract, another oil from grape seeds, usually a by-product of wine production, is also used as a cosmetic ingredient, as well as in marinades, infusions, and dressings in cooking [204].

Recent efforts have focused on incorporating phenolic compounds into commercial products. The peel and skin of the pomegranate have been identified as a source of ellagitannins and punicalagins, which have been patented for their commercial use as antioxidants in food and cosmetic products [205]. Microencapsulated polyphenols from pomegranate peel have been used to enrich ice cream with natural antioxidants [206], and bread has been fortified with pomegranate peel powder [207]. Tomato pomace is most commonly used in powdered form as a nutritious and antioxidant additive in various foods, including wheat flour products, meat products, dairy and oil products, and confectioneries [208]. Abid et al. [209] enriched Tunisian butter with an extract from tomato processing by-products. The storage stability of the enriched butter during 60 days of storage at 4 °C significantly improved due to the strong antioxidant activity, which inhibited the formation of peroxides and conjugated dienes, as well as the degradation of unsaturated fatty acids to oxidation products. The addition of tomato by-products not only improves the nutritional profile of dairy products but also contributes to their sensory qualities by giving them a unique flavor and color [210]. Large quantities of potatoes are processed for human consumption, resulting in significant amounts of waste in the form of potato peels. These peels can serve as a potential source of fiber in processed products [211]. Along with fiber, the peels are rich in antioxidants and essential nutrients, with a potential for implementation in various formulations. Potato peel powder in baked goods can enhance their nutritional value while improving texture and moisture retention [212]. Franco et al. [213] found that antioxidant protection and inhibition of hexanal production in soybean oil increased with higher concentrations of potato peel extract. Phenolic acids are the most important compounds found in potato peels, with chlorogenic acid (49–61%) being the most abundant, followed by caffeic acid (2.3–19.9%), gallic acid (7.8%), and protocatechuic acid (0.21%) [214].

Anthocyanins are utilized in the food industry as a natural alternative to synthetic dyes [215]. In a study by Ali et al. [216], natural anthocyanins extracted from red onion skins were successfully applied as colorants in confectioneries, especially in hard candies and glazed jellies. The products containing 0.3% anthocyanins in hard candies and 0.25% in glazed jelly received high ratings for color and overall acceptability, comparable to those achieved with synthetic dyes. Additionally, the use of natural colorants meets the growing consumer demand for clean label products, which focus on transparency and natural ingredients [214].

Oilseed by-products, such as sunflower seeds, soybean seeds, soybean oil waste, soybean-processing wastewater, olive pomace, and olive oil-processing wastewater, also contain significant amounts of bioactive compounds [217]. These include phytosterols, polyphenols, and proteins [218]. The by-products resulting from the various oil productions are rich in proteins and fibers and contain numerous other bioactive compounds, which can be incorporated into the production of functional bakery products [219]. The addition of oilseed cakes to bakery products increases the sustainability of production. Oilseed cakes, especially those leftover after the production of cold-pressed and virgin oils, have a high nutritional value (flavonoids, proanthocyanins, and phenolic acids) [220]. By adding up to 15% of these meals to bakery products, functional products with greater added value and good sensory and technological properties can be obtained [221]. Due to the high oil content, special attention should be paid to the storage conditions and shelf life of the meals and the products [222].

Whey, a by-product of cheese production, is an important waste product in the dairy industry. For every 10 kg of cheese produced, up to 9 kg of whey is generated [223]. It is generally used as fertilizer, animal feed, and for human consumption. Whey contains approximately 55% of the nutrients in milk and represents about 20% of the total protein content, which corresponds to 85 to 95% of the original milk volume. Whey is the serum phase of milk, the liquid remaining after the removal of fats and casein, and is composed mainly of soluble components such as lactose, soluble salts, and globular proteins. Liquid whey is characterized by its greenish-yellow hue, which is attributed to the presence of riboflavin (vitamin B2) [224]. The nutritional profile and technological properties of whey make it a valuable ingredient in various food products and nutraceutical applications. Studies have highlighted its potential health benefits, including its role as a source of high-quality protein and bioactive compounds that may enhance muscle recovery and support immune function [225]. Furthermore, the rich chemical composition of whey presents opportunities for its utilization in functional foods, helping to reduce and promote sustainability in the dairy sector [226].

Considering the nutritional and chemical properties, antioxidant activity, and present BACs, the coffee bean silver skin that is left over after roasting appears promising for use in the food industry [227]. Due to its fiber-rich composition, silver skin has been incorporated into baked goods as a source of dietary fiber [228] and used in the production of a novel beverage for weight control [229]. Moreover, extracts from silver skin could serve as a natural colorant and fiber source in biscuits, which could improve the quality, shelf life, and sensory properties of biscuits [230]. The bioactive potential of coffee silver skin also makes it a viable alternative to synthetic chemicals in cosmetic formulations. Its rich content of phenolic compounds, melanoidins, and caffeine contributes to its strong antioxidant capacity, which can be used against aging and shows resistance to oxidative stress induced by tert-butyl hydroperoxide in human keratinocytes [231]. The valorization of food waste as a source of bioactive compounds and functional ingredients represents a significant shift toward sustainable food systems. By repurposing waste such as fruit peels, pomace, and dairy by-products, the food industry can reduce environmental burdens while enhancing product nutrition and shelf life. However, the practical application of food waste in food products faces several challenges. First, variability in the composition and quality of by-products complicates standardization and consistency in formulations. Moreover, many of these waste streams are highly perishable, requiring energy-intensive stabilization methods like drying or freeze-drying, which can be economically and environmentally costly. Regulatory hurdles and consumer perceptions also limit widespread adoption. While natural additives are appealing, acceptance may waver if products are linked to waste. Despite these obstacles, evidence supports the efficacy of food waste-derived ingredients, such as polyphenols from fruit peels or proteins from whey, for improving antioxidant activity and nutritional profiles. Advancements in processing technologies and clear labeling practices will be crucial to overcoming skepticism and achieving commercial viability. Ultimately, while promising, the integration of food waste into food products demands a careful balance between functionality, safety, economic feasibility, and consumer trust.

## 4. The Valorization of Food Waste into Nutraceuticals and Dietary Supplements

The valorization of food waste into nutraceuticals and dietary supplements has garnered increasing global attention as a promising solution to address both environmental sustainability and public health goals [232]. This innovative strategy not only supports the principles of a circular economy by turning waste into value-added products but also aligns with the United Nations Sustainable Development Goals (SDGs), particularly those targeting responsible consumption and production (SDG 12), good health and well-being (SDG 3), and climate action (SDG 13) [233]. In recent years, a growing body of research has investigated the bioactive potential of food waste streams. For example, grape pomace, a major by-product of the winemaking industry, is a rich source of resveratrol and quercetin, two powerful polyphenolic compounds. Resveratrol has been extensively studied for its cardioprotective, neuroprotective, and anti-aging effects, while quercetin exhibits potent antioxidant and anti-inflammatory activity [234]. Likewise, citrus peels, commonly discarded during juice production, contain high concentrations of hesperidin and naringin, flavonoids known to support vascular health and reduce oxidative damage [235]. Another illustrative case is banana peels, which are rich in catechins and anthocyanins. These compounds have shown potential in mitigating oxidative stress and regulating glucose metabolism, making them candidates for managing conditions such as diabetes and metabolic syndrome [236]. Similarly, apple peels and other fruit skin residues are abundant in quercetin glycosides and chlorogenic acid, with antioxidant activities comparable to that of synthetic antioxidants like ascorbic acid (vitamin C) [237]. These examples underscore how food waste, often discarded at an industrial scale, can be reimagined as a sustainable source of health-promoting ingredients.

Vegetable waste, including peels and trimmings from carrots, beets, tomatoes, and leafy greens, has also shown promise. Tomato skins, for instance, contain lycopene, a carotenoid with strong antioxidant and anti-proliferative properties, which is now being incorporated into capsules and supplements aimed at reducing prostate cancer risk and improving skin health [238]. Carrot waste is similarly valued for its β-carotene content, a precursor of vitamin A that plays a vital role in immune function and vision.

Technological advancements in extraction and processing have been pivotal in realizing the full potential of these by-products. Despite these advancements, several challenges hinder the wide-scale application of food waste-derived nutraceuticals. One primary concern is the variability in the composition of food waste, which depends on several factors, including plant variety, geographic origin, harvesting conditions, and post-harvest handling. This variability can affect the consistency and reproducibility of the bioactive compounds extracted, posing challenges for both quality control and regulatory approval [239]. Additionally, the presence of contaminants such as pesticides, heavy metals, or microbial pathogens in food waste raises safety concerns [240]. It is critical to implement robust decontamination and purification steps and adhere to strict food safety guidelines to ensure that the end products are safe for human consumption. Furthermore, the regulatory framework for nutraceuticals varies significantly across regions, complicating the commercialization process. In the EU, for instance, compounds classified as novel foods must undergo a rigorous approval process, whereas in the US, the FDA requires compliance with the Dietary Supplement Health and Education Act (DSHEA) but does not mandate pre-market approval unless new dietary ingredients are used. Another limitation lies in consumer acceptance. Although the concept of food waste-derived products is sustainable and scientifically sound, some consumers may harbor negative perceptions or misconceptions about consuming supplements sourced from waste [241]. Clear labelling, transparent communication, and public education campaigns are essential to increase awareness and acceptance of these products. From a scientific standpoint, further research is needed to fully understand the bioavailability, metabolism, and long-term health effects of food waste-derived bioactives. While in vitro and animal studies have demonstrated promising results, there is a scarcity of well-designed clinical trials that confirm their efficacy and safety in humans. Moreover, synergistic effects among bioactive compounds are often overlooked, despite growing evidence that combinations of phytochemicals can exert greater therapeutic effects than isolated constituents [242].

The integration of food by-product valorization into nutraceutical development also has significant economic and environmental benefits. By reducing waste disposal costs and generating new revenue streams, food processors can improve profitability. Additionally, repurposing waste into valuable supplements contributes to greenhouse gas reduction, decreased reliance on synthetic additives, and improved resource efficiency [243]. To accelerate progress in this area, collaborative efforts between academia, industry, and regulatory bodies are essential. Research institutions can lead the way in identifying new bioactives and optimizing extraction processes, while industry partners can scale up production and manage supply chains. Policymakers, meanwhile, can foster innovation by providing clear regulatory pathways and financial incentives for sustainable practices.

## 5. Challenges and Future Perspective

Food waste from the processing of fruits, vegetables, grains, and other agricultural raw materials, such as peels, skins, seeds, stems, and leaves, is usually considered inedible [244], but the nutritional value of these by-products demonstrate that industry has an opportunity to rethink the use of food waste [245]. These materials can be harnessed to develop new and functional food products, dietary supplements, or to enhance the nutritional profile of existing products [246]. Repurposing these materials for human consumption can contribute to reducing food waste while simultaneously providing the market with novel, health-promoting ingredients. However, in order to use these by-products and successfully introduce such products to market, it is essential to align with regulatory and legislative policies [247,248] to ensure that they meet food safety standards, proper labeling requirements, and environmental sustainability criteria [249]. Regulatory bodies such as the European Food Safety Authority (EFSA) and the Food and Drug Administration (FDA) require extensive testing and validation to demonstrate that food waste, when incorporated into food products, is safe for consumption [250,251]. This includes assessing the presence of any potential contaminants, allergens, or other harmful substances that could arise from the processing of a by-product [252].

The food waste that can be used as an additive or supplement and is rich in natural antioxidants often presents a complex mixture of secondary metabolites, many of which may not be fully identified [253]. This complexity poses a challenge in terms of understanding their complete composition and functionality once digested. One of the key issues with natural extracts is the variability in both quality and composition. They can be influenced by plant variety [254], climatic conditions [255], and the degree of ripeness at the time of harvest [256]. As a result, it becomes essential to analyze the composition of each extract batch to ensure consistency and, if necessary, adjust the quantity added to the final product [257]. The problem of variability in natural extracts highlights the need for advanced quality control and standardization practices. Without such measures, the effectiveness of antioxidants can fluctuate, leading to inconsistent product properties [258,259,260]. It may also influence sensory properties, depending on the form of waste and the product in which it is used [261]. To reduce the negative impact of natural extract variability, a fair compromise is to combine natural and synthetic antioxidants to achieve a synergistic effect [262].

Alongside the inconsistent chemical composition, utilizing BACs from food waste in the industry is significantly limited due to high contamination risks [263], including pesticide residues [264], mycotoxins [265], microbial contaminants, and heavy metals [266]. Thus, safety assessments are essential to ensure that potentially reusable food waste and derived extracts are suitable for bioactive molecule extraction. To address these contamination risks, food safety protocols require comprehensive testing at various stages, from raw material collection to the final extract [267].

The processes for reducing these antinutrients, while effective, can significantly increase operational costs, energy consumption, and processing time, particularly in large-scale production [268]. A promising alternative for industrial applications for removing contaminants from wastewater streams derived from processing raw materials is the use of membrane processes [269]. These methods are particularly valuable for recovering, separating, and fractionating specific bioactive compounds [270]. Their application has seen particular success in the valorization of food by-products and the treatment of wastewater in the food sector, making them an increasingly adopted solution for industries seeking to integrate sustainable practices and maximize resource recovery.

Due to the heterogeneity and complex biochemical structure of food waste, it is a challenge to utilize it in a single process [271]. The difficulties associated with using a single production method to maximize the value of food waste frequently lead to the development of integrated production systems, which enable the generation of multiple products through a targeted valorization strategy [272]. To promote the utilization of agro-industrial food waste for the development of new products, several steps must be optimized, including preprocessing, fermentation processes or extraction, and subsequent treatment [273].

**Figure 6:**
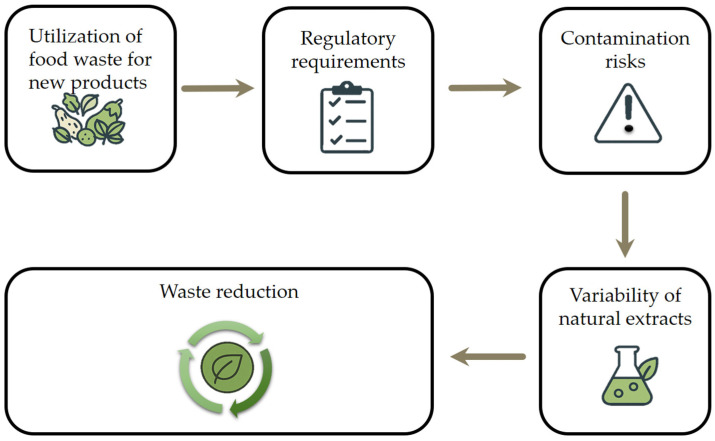
Challenges and future perspectives in waste management.

Hodges et al. [274] highlighted the significant challenges that lie ahead in feeding the projected global population of nine billion people by 2050 [275]. Meeting future demand will be difficult due to the compounded effects of climate change and the depletion of natural resources [276]. These factors restrict agricultural growth and limit food production, creating significant obstacles for food safety [277]. Additionally, the inefficiencies in the food supply chain, particularly food thrown away, contribute to the problem. Tomlinson [277] estimated that the food waste could be sufficient to feed 1 billion people annually. A shift toward circular economy principles, where food by-products and waste are repurposed for human consumption or industrial use, presents a promising way of reducing the food waste created by industry. Figure 6 outlines key considerations in the utilization of food waste for new product development. It emphasizes the importance of meeting regulatory requirements and addressing contamination risks and the variability of natural extracts, which are critical challenges in ensuring product safety and consistency. Despite these hurdles, the approach ultimately contributes to significant waste reduction and supports sustainable innovation.

In the hierarchy of waste management, waste prevention and minimization remain the priorities [278], followed closely by the utilization of by-products, especially for human consumption [279]. Achieving zero waste in the food industry remains a challenge, requiring efforts from industry, governmental institutions, academia, individuals, and organizations [280,281,282].

## 6. Conclusions

The valorization of food waste represents a strategic and sustainable approach to mitigating the global challenges of food loss, environmental degradation, and resource inefficiency. By reclassifying food by-products as sources of valuable bioactive compounds, particularly natural antioxidants and functional polysaccharides, the food and allied industries can simultaneously reduce waste and develop products with added nutritional and functional value. These bioactives have demonstrated significant potential in reducing oxidative stress and preventing chronic conditions, such as cardiovascular and neurodegenerative diseases. The application of green and efficient extraction techniques, such as enzymatic hydrolysis and ultrasound-assisted extraction, further enhances the recovery of these compounds while preserving their structural integrity and bioactivity. As such, food waste valorization not only aligns with circular economy principles but also contributes to the advancement of clean-label, health-promoting ingredients. This review underscores the importance of integrating scientific innovation with sustainability goals to foster a resilient and health-oriented food system. Continued research into optimized extraction methods and the biological efficacy of recovered compounds is essential to fully realize the potential of food by-product utilization. In this context, food waste management becomes not merely a challenge but also a valuable opportunity to support human health and environmental protection through the development of natural antioxidant-rich formulations.

## Figures and Tables

**Table 1:** Comparison of extraction techniques for recovery of bioactive compounds from food waste [21,22,23,24,25].

Extraction Method	Principle	Solvent Use	Time and Energy Efficiency	Selectivity/Yield	Preservation of Bioactivity	Environmental Impact	Key Advantages	Key Limitations
Conventional Solvent Extraction (CSE)	Organic solvents extract compounds via diffusion	High (organic solvents, e.g., ethanol, methanol)	Low; long time, high energy	Moderate; depends on polarity and solvent used	May degrade heat-sensitive compounds	High; solvent toxicity, waste issues	Low cost, simple, well-established	Low efficiency; high solvent consumption; not eco-friendly
Ultrasound-Assisted Extraction (UAE)	Cavitation bubbles enhance cell disruption and mass transfer	Moderate (often ethanol-water mixtures)	High; short extraction time	High; enhances extraction of phenolics and flavonoids	Good if temperature is controlled	Lower than CSE; greener solvents	Cost-effective, scalable, gentle on bioactives	Equipment scale-up challenges; needs parameter optimization
Microwave-Assisted Extraction (MAE)	Dielectric heating causes rapid internal heating and cell rupture	Moderate to low	Very high; rapid heating	High for polar compounds	Good under controlled exposure	Low, especially with water or green solvents	Fast, solvent-saving, suitable for thermolabile compounds	Risk of degradation at high temps; special equipment required
Subcritical Water Extraction (SWE)	Water at 100–374 °C under pressure acts as polar–nonpolar solvent	Water only	Moderate to high; pressure/temperature dependent	High; extracts wide range of bioactives	High for heat-stable compounds	Very low; no solvent residues	Green process, non-toxic, broad solubility range	High-pressure setup; limited to heat-stable compounds
Supercritical CO_2_ Extraction (SC-CO_2_)	CO_2_ above critical point acts as a solvent (gas-like diffusivity, liquid-like solvency)	CO_2_ (non-toxic, recyclable); may use co-solvents (e.g., ethanol)	High; rapid mass transfer	High for non-polar bioactives (e.g., lipids, carotenoids)	Excellent for thermolabile compounds	Very low; no toxic residues	Non-toxic, solvent-free products, selective extraction	High equipment cost; limited for polar compounds unless co-solvents used
Pulsed Electric Fields (PEF)	Short pulses of high-voltage electric fields permeabilize cell membranes	Aqueous medium	Very high as pre-treatment	Enhances extraction when combined with other methods	Excellent for sensitive bioactives	Low; no chemicals used	Non-thermal, preserves nutrients, reduces solvent use	Not a stand-alone extraction; needs coupling with other methods
Deep Eutectic Solvents (DESs)	Natural compounds (e.g., sugars, acids) form liquid mixtures for selective solubilization	Low toxicity; biodegradable	High; depends on DES formulation	High selectivity; good for phenolics, flavonoids	Very good; mild conditions	Very low; customizable and biodegradable	Tunable, green solvents; excellent for polyphenol extraction	Post-extraction separation/purification can be complex

**Table 2:** Antioxidant activity of the polyphenols and flavonoids extracts from food waste. Antioxidant activity was assessed using widely recognized methods, including DPPH, ABTS, and FRAP assays, to provide a comprehensive evaluation of the antioxidant potential.

Food Waste	Extraction Method	Extraction Solvent	Polyphenols/Flavonoids Concentration Antioxidant Activity	Reference
*Moroccan* *cannabis* stem 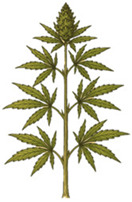	Ultrasound-assisted extraction	Ethanol, methanol, acetone, and water	Acetone: TPC = 99 ± 22.82 mg GAE/g, TFC = 117.33 ± 24.82 mg QE/g, DPPH-IC50 = 22.13 ± 4.32 µg/mL Ethanol: TPC = 95.34 ± 21.14 mg GAE/g, TFC = 98.57 ± 23.17 mg QE/g, DPPH-IC50 = 19.62 ± 3.56 µg/mL Methanol: TPC = 86.32 ± 19.32 mg GAE/g, TFC = 89.65 ± 19.87 mg QE/g, DPPH-IC50 = 26.00 ± 3.24 µg/mL Water: TPC = 66.07 ± 1.23 mg GAE/g, TFC = 62.88 ± 4.64 mg QE/g, DPPH-IC50 = 45.09 ± 3.24 µg/mL	[21]
Orange peel 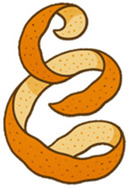	Classic solid–liquid extraction	Ethanol, methanol, and water	Ethanol: TPC = 1.84 ± 0.05 mg GAE/g, TFC = 74.01 ± 5.1 mg QE/g, DPPH-IC50 = 55.5 ± 2.15%, FRAP = 9.6 ± 0.3 mg TE/g, ABTS = 6.5 ± 0.25 µmol TE/g Methanol: TPC = 1.89 ± 0.05 mg GAE/g, TFC = 80.05 ± 5.2 mg QE/g, DPPH-IC50 = 58.5 ± 2.5%, FRAP = 11.9 ± 2.3 mg TE/g, ABTS = 7.2 ± 0.35 µmol TE/g Water: TPC = 1.56 ± 0.04 mg GAE/g, TFC = 50.5 ± 4.2 mg QE/g, DPPH-IC50 = 42.8 ± 1.85%, FRAP = 8.9 ± 0.2 mg TE/g, ABTS = 5.45 ± 0.1 µmol TE/g	[38]
Olive pomace 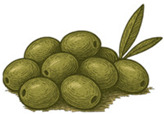	Classic solid–liquid extraction	Natural deep eutectic solvents	TPC = 15.56 mg GAE/g dw, FRAP = 178.14 mol FSE/g dw, DPPH = 72.75 mol TE/g dw	[39]
Potato peel 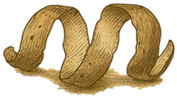	Classic solid–liquid extraction	Ethanol	TPC = 2.04 ± 0.87 mg GAE/g dw, TFC = 0.21 ± 0.008 mg QE/g dw DPPH = 179.75 ± 3.18 μg/mL	[40]
Spring onion leaves 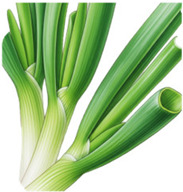	Microwave-assisted extraction	Ethanol	TPC = 1.35 mg GAE/g dw FRAP = 14.02 mmol Fe(II)/g dw	[41]
Tomato waste 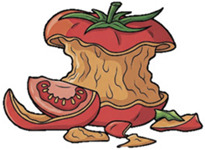	Ultrasound assisted extraction	70% (*v*/*v*) ethanol–water	TPC = 4.63 ± 0.016 mg GAE/g, TFC = 0.956 ± 0.07 mg RUE/100 g, ABTS = 27.90 ± 0.10 µmol TE/g	[42]

TPC = total phenols content, TFC = total flavonoids content, DPPH = 2,2-diphenyl-1-picrylhydrazyl; FRAP = Ferric Reducing Antioxidant Power; ABTS = 2,2-Azino-bis-(3-ethylbenzothiazoline-6-sulfonic acid) diammonium salt; TE = Trolox equivalent; QE = quercitine equivalents; FSE = ferrous sulfate equivalents; GAE = gallic acid equivalents; RUE—rutin equivalents; dw = dry weight.

**Table 3:** Antioxidant activity of carotenoid extracts from food waste.

Food Waste	Extraction Method	Extraction Solvent	Antioxidant Activity	Reference
Peach pomace 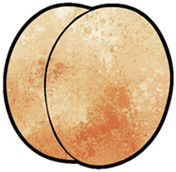	Ultrasound-assisted enzymatic extraction	Hexane/acetone/ethanol; 50/25/25 *v*/*v*	ABTS = 1933.33 mg Trolox/L FRAP = 52.66 µmol Trolox/L	[52]
Carrot pomace 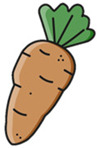	Ultrasonication and high shear dispersion techniques	Flaxseed oil	ABTS = 1596.04 ± 69.45 μg Trolox/mL DPPH = 380.21 ± 39.62 μg Trolox/mL FRAP = 941.20 ± 19.91 μM Trolox/mL	[53]
Passion fruit peel 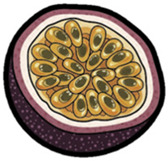	Ultrasound-assisted extraction	Olive oil and sunflower oil	Inhibition of DPPH = 35.2 ± 1.4%	[54]
Carrot juice 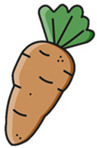	Microwave-assisted extraction	Flaxseed oil	Inhibition of DPPH = 70.67 ± 0.85%	[55]
Tomato waste 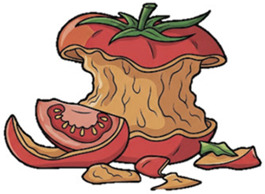	Conventional solid–liquid extraction at room temperature	Hydrophobic deep eutectic solvents composed of menthol and fatty acids	*A*_AR_ = 63.7 ± 4 µmol AAE/g dw *P*_R_ = 26.7 ± 1.8 µmol AAE/g dw	[56]
15 food waste matrices 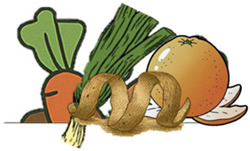	Supercritical fluid extraction	Supercritical CO_2_ and ethanol as co-solvent	SPF = 36.6 ± 2.0%, SPP = 20.7 ± 1.8%, TMF = 30.4 ± 1.7%, TMP = 87.9 ± 1.6%, APF = 39.2 ± 2.2%, APP = 51.9 ± 2.2%, PKF = 42.3 ± 1.1%, PKP = 77.1 ± 1.0%, PCF = 7.0 ± 5.1%, PCP = 34.1 ± 3.1%, GPF = 17.5 ± 4.6%, YPF = 49.7 ± 0.9%, RPF = 46.5 ± 2.7%, XPW = 19.0 ± 3.4%, MIX = 57.7 ± 2.5%	[57]
Orange peel 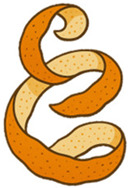	Ultrasound-assisted extraction	Organic solvent, vegetable oils, fatty acids, and deep eutectic solvents	Hexane: DPPH = 944.3 ± 13.1 µM TE/mL, ABTS = 355.1 ± 4.5 µM TE/mL, FRAP = 854.2 ± 8.5 µM TE/mL Olive oil: DPPH = 1790.6 ± 17.7 µM TE/mL, ABTS = 661.2 ± 4.8 µM TE/mL, FRAP = 990.6 ± 11.4 µM TE/mL Octanoic acid: DPPH = 2438.8 ± 21.2 µM TE/mL, ABTS = 463.1 ± 6.5 µM TE/mL, FRAP = 743.41 ± 2.1 µM TE/mL Octanoic: L-Proline DES; DPPH = 1340.4 ± 18.3 µM TE/mL, ABTS = 1057.3 ± 18.3 µM TE/mL, FRAP = 1456.4 ± 12.2 µM TE/mL	[58]
*Citrus sinensis* peels 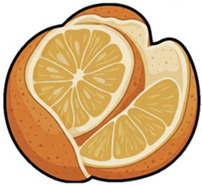	Ultrasound-assisted extraction	Hydrophobic deep eutectic solvents	Methanol: Eucalytol DES; DPPH ≈ 2500.1 ± 5.3 µM TE/mL	[59]
Pumpkin peel 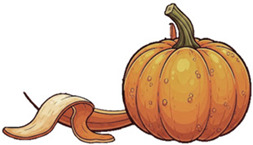	Ultrasonic-assisted extraction	3:1 *v*/*v* n-hexane/acetone solvent mixture	DPPH = 7.25 µM TE/g dw	[60]
Cantaloupe waste 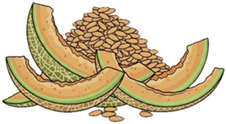	Ultrasound-assisted extraction	Hexane/acetone mixture	IC_50_ = 7.33 ± 0.22 μg/mL	[61]

*A*_AR_ = antiradical activity; *P*_R_ = reducing power; AAE = ascorbic acid equivalents; DPPH = 2,2-diphenyl-1-picrylhydrazyl; FRAP = ferric-reducing antioxidant power; ABTS = 2,2-Azino-bis-(3-ethylbenzothiazoline-6-sulfonic acid) diammonium salt; SPF = sweet potato flesh; SPP = sweet potato peels; TMF = tomato flesh; TMP = tomato peels; APF = apricot flesh; APP = apricot peels; PKF = pumpkin flesh; PKP = pumpkin peels; PCF = peach flesh; PCP = peach peels; GPF = green pepper flesh; YPF = yellow pepper flesh; RPF = red pepper flesh; XPW = pepper wastes; MIX = sample mix; TE = Trolox equivalent; dw = dry weight.

**Table 4:** Overview of vitamins in food waste and their health benefits.

Solubility	Vitamin	Structure	Properties
Fat-soluble vitamins	**A**	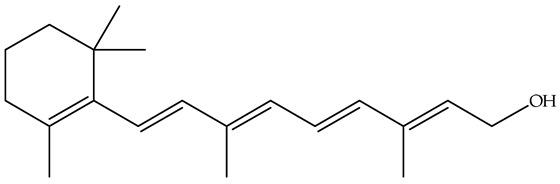	Vitamin A, which is abundant in vegetable peels and scrapes such as carrot tops and pumpkin seeds, is crucial for vision, immune function, cell growth, and differentiation [60,76]. Its deficiency can lead to night blindness, impaired immune response, and even blindness [77]. The carotenoids present in these food wastes can be converted into vitamin A in the body and are, therefore, a valuable source of this essential nutrient.
	**D**	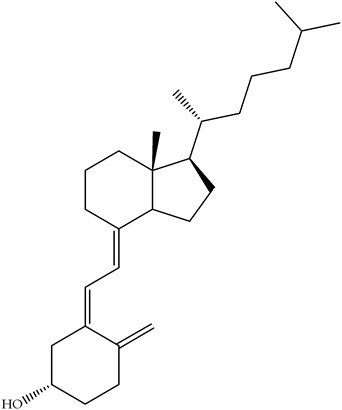	Although vitamin D is primarily synthesized in the skin through sun exposure, it can also be obtained from food. Some food waste, particularly from the processing of fatty fish, may contain vitamin D [78]. This vitamin is important for calcium absorption, bone health, and immune function. Vitamin D deficiency is linked to rickets in children and osteoporosis in adults, as well as an increased risk of infections and other health problems [79].
	**E**	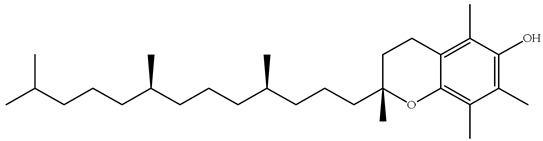	Vitamin E is found in various food wastes, including seed husks and vegetable oils, and acts as a powerful antioxidant that protects cells from free radical damage. It also plays a role in immune function and blood clotting [80,81]. A deficiency is relatively rare but can lead to neurological problems.
	**K**	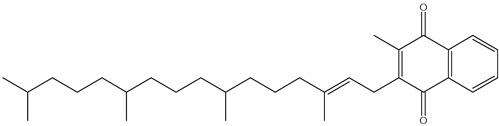	Vitamin K is found in the waste of green leafy vegetables and some fruit peels [82] and is essential for blood clotting and bone health. It also plays a role in regulating calcium metabolism. A deficiency of vitamin K can lead to bleeding disorders and impaired bone health.
Water-soluble vitamins	**B**	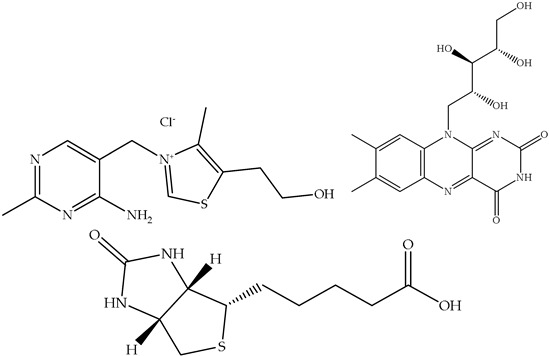	This group of vitamins plays a crucial role in energy metabolism, DNA synthesis, and nerve function. Various B vitamins are found in different food wastes [83,84]. For example, B vitamins such as folate are found in vegetable leaves. B vitamins are important for converting food into energy, supporting nerve function, and the formation of red blood cells. A deficiency of B vitamins can lead to a number of health problems, including fatigue, anemia, and neurological problems.
	**C**	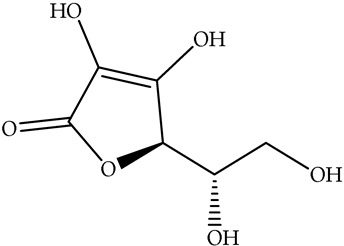	Vitamin C is abundant in citrus fruit peels and other fruit and vegetable waste and is a powerful antioxidant that supports immune function, collagen synthesis, and wound healing [85,86,87]. It also promotes the absorption of iron. A deficiency of vitamin C can lead to scurvy, which is characterized by fatigue, weakness, and bleeding gums.

**Table 5:** Overview of some applications of food waste in food products.

Food Waste/Source	Bioactive Compounds	Application	Positive Effect	References
**Grape seed oil**	Proanthocyanidins Phenolic compounds Vitamin E isomers (ɣ-tocotrienol)	Culinary oil Addition to meat products	Enhancing lipid stability of meats Protection against oxidative brain damage Antimicrobial activity	[127,128,129,130,131]
**Rice husk and bran**	δ-Tocopherols, ɣ-oryzanol,	Supplement	Reduction in serum levels of hs-CRP and IL-6 (anti-inflammatory effect)	[132,133,134]
**Olive oil wastewater**	Hydroxytyrosol Verbascoside Oleuropein Ligstroside	Nutraceutical Supplement Stock feed	Tumor cell inhibition in vitro and in vivo Natural cheese antioxidants by adding to sheep feed	[135,136]
**Banana peel**	Dietary fiber Galleocatehin Anthocyanins	Herbal medicine Implementation in food products (noodles) Supplementation	Reducing blood sugar Bacteriostatic or fungistatic	[137,138,139,140,141]
**Carrot peel**	Carotenoids Xantophylls	Addition to jams Implementation in bakery products	Increasing antioxidant activity of products Increasing the amount of dietary fibers	[142,143,144,145]
**Cucumber peel**	Cucurbitacins Cucumegastigmanes I and II Cucumerin A and B	Development of food and snack films	Shelf-life extension of cheese, more stable pH, moisture level, and lipid oxidation, sensory improvements	[146,147,148]
**Barley bran**	Beta glucan Arabinoxylan	Edible films	Lowering serum cholesterol Acceleration of gastrointestinal transit time	[149,150]
**Mango peel**	Carotenoids Dietary fiber	Mango peel powder incorporation	Increase in dietary fiber and polyphenols (0.46 to 1.80 mg/g in macaroni)	[151,152,153,154]
**Peanut shell**	Luteolin Isosaponeratin	Addition to peanut butter	Increasing fiber levels and antioxidants in peanut butter	[155]
**Guava peel**	Lycopene Beta carotenes	Flour	Replacing wheat flour in cookie preparation—decreasing fat and carbohydrate levels	[156]
**Avocado peel**	5-O-caffeoylquinic acid Catechin Epicatechin	Extract	Prevention of oxidation in meat Antimicrobial activity against *Listeria innocou* and *E. coli*	[157,158,159]
**Apple peel**	Cyanidin 3-glucoside	Extract	Inhibition of HepG2 human liver cancer cells Strawberry preservation with chitosan-based coating Improvement of oxidative stability of mayonnaise	[160,161,162]
**Olive leaves**	Oleuropein Tyrosol Hydroxytyrosol Quercetin Rutin	Substrate for fermentation	Production of kombucha with infused olive leaves—increased antioxidant activity and sensory properties	[163]
**Pumpkin flour (cold-pressed cake)**	Beta-carotene	Addition to products	Fortification of sponge cake	[164]
**Wheat germ**	Carotenoids y-oryzanol biogenic amides	Addition to products	Improvement of sensory and antioxidative properties	[165,166]
**Broccoli waste**	Glucosinolates Isothiocyanates Vitamin C		Novel green tea-based beverage with added nutritional value	[167,168]
**Animal blood**	Hemoglobin Thrombin ACE-inhibiting bioactive peptides		Stabilizer Color additive Emulsifier Protein and iron supplement	[169,170,171,172]
**Fish oil**	Omega-3 fatty acids	Extract	Food supplements	[173,174]
**Wine pomace**	Anthocyanins	Extract Flour	Colorant food additive in yogurt and salad dressings Antioxidative activity Development of functional foods	[119,175,176,177]
**Eggshells**	Calcium		Development of functional foods	[178,179,180]
**Algae**	Alginate Omega-3 fatty acids	Residual biomass	Implementation in active edible packaging	[161,181]
**Whey**	Protein		Supplements Addition to products	[182,183]
**Artichoke by-product**	Chlorogenic acid Cynarin Narirutin	Extract	Increase in antioxidant activity and shelf life in tomato juice	[184,185]
**Raspberry pomace**	Gallic acid Caffeic acid Ellagic acid Fiber	Dried pomace	Addition to cookies, increase in fibers and organoleptic properties	[186,187,188]
**Coconut skin milk**	Protein	Coconut protein powder	Emulsifying properties	[189]
**Cauliflower trimmings**	Dietary fibers Carotenoids Vitamin E	Ready-to-eat extrudes	Increased protein content and water absorption index	[190]

## Data Availability

All data are presented in the manuscript.
